# The Cannabis Research Image Database (CRESIDA): A standardized and validated image set for studying cannabis cue reactivity

**DOI:** 10.1111/add.70516

**Published:** 2026-06-28

**Authors:** Janna Cousijn, Emese Kroon, Chao Suo, Tom P. Freeman, Chandni Hindocha, Marianna Quinones‐Valera, Valentina Lorenzetti

**Affiliations:** ^1^ Neuroscience of Addiction (NofA) Lab, Center for Substance Use and Addiction Research (CESAR), Department of Psychology, Education & Child Studies Erasmus University Rotterdam Rotterdam the Netherlands; ^2^ QIMR Berghofer Herston Queensland Australia; ^3^ Neuroscience of Addiction and Mental Health Program, Healthy Brain and Mind Research Centre, Mary McKillop Institute, School of Behavioural & Health Sciences, Faculty of Health Science Australian Catholic University Melbourne Victoria Australia; ^4^ Turner Institute for Brain and Mental Health, School of Psychological Sciences & Monash Biomedical Imaging Facility Monash University Clayton Victoria Australia; ^5^ School of Psychology University of Queensland St Lucia Queensland Australia; ^6^ Addiction and Mental Health Group, Department of Psychology University of Bath Bath UK; ^7^ Clinical Psychopharmacology Unit, Research Department of Clinical, Educational & Health Psychology University College London London UK

**Keywords:** cannabis, cannabis use disorder, cue reactivity, image database, image repository, tobacco

## Abstract

**Background and aims:**

Cannabis cue reactivity paradigms are instrumental in studying the behavioral and neurocognitive mechanisms of cannabis use and cannabis use disorders; however, image sets used for cannabis cue reactivity paradigms vary between studies, and the lack of reliability and validity assessment hinders the quality of evidence they generate. The main aim of this study was to create a novel, open access, standardized and representative database of cannabis use‐related images including control images matched by resolution, luminosity and complexity: The Cannabis Research Image Database (CRESIDA). The secondary aim was to examine whether subjective cannabis cue‐induced craving was associated with cannabis use severity and whether this relationship was moderated by image type. As an illustrative example of how our open data can be used and how sample characteristics can shape cue reactivity, we also explored the role of cannabis–tobacco mixing by comparing cannabis cue induced cannabis and tobacco craving between individuals who did and did not mix the substances.

**Design:**

An online survey was administered to participants recruited via online platforms, community advertisement and snowballing.

**Setting:**

USA, the Netherlands and Australia.

**Participants/cases:**

689 participants who consumed cannabis monthly to daily (385 men, 298 women, 6 other) were recruited between January 2022 and May 2024.

**Measurements:**

Out of 93 cannabis images and 93 matched neutral images, participants each rated 31 image pairs for cannabis craving (the primary outcome), arousal, valence and tobacco craving. Participants were characterized for socio‐demographic data, level of cannabis use and related problems and mixing cannabis and tobacco. A subset of 78 images was selected for further analysis based on cannabis craving results. Image ratings were evaluated for internal consistency (α). Furthermore, we examined the association between cannabis cravings and cannabis use characteristics, and explored if cannabis craving ratings were affected by image type (i.e. product, paraphernalia and actions) and by using cannabis alone vs. mixing cannabis and tobacco.

**Findings:**

The database showed excellent reliability (α = 0.995–0.965). Cannabis craving, valence and arousal discriminated cannabis and control images. More cannabis use days [unstandardized beta (β) = 0.162, *P* < 0.001] and cannabis use‐related problems (β = 0.268, *P* < 0.001) were statistically significantly associated with higher image‐related cannabis craving. Mixing cannabis with tobacco, compared with using cannabis alone, was associated with the presence of tobacco craving in relation to cannabis images, and with greater cannabis craving in relation to cannabis images (β = −0.457, *P* < 0.001).

**Conclusions:**

Images in the open access Cannabis Research Image Database (CRESIDA, https://osf.io/dc9nz/) appear to be reliable and valid for the scientific study of cue reactivity internationally, providing a broad range of free to use cannabis and control images.

## INTRODUCTION

Cannabis cue reactivity paradigms are widely used to study the mechanisms underlying cannabis use and cannabis use disorder (CUD) [1, 2, 3]. During such paradigms, participants are typically presented with cannabis‐related visual cues, though tactile, auditory, gustatory, olfactory or multisensory cues have also been used [1]. In individuals who consume cannabis, exposure to cannabis cues, relative to control cues, can trigger multiple behavioral (e.g. self‐reported craving, attentional bias, approach bias [1, 4]), physiological (e.g. higher heart rate, higher skin conductance [1]) and neural (e.g. greater brain activity in fronto‐limbic motivation‐related brain areas [2, 3]) responses. These multi‐modal responses to cannabis cues are thought to reflect an individual’s motivation to use cannabis [5, 6]. Cannabis cue‐induced craving and activity in motivation‐related brain areas is generally higher in those individuals who use cannabis more heavily and who experience more severe cannabis use‐related problems, including CUD [2, 3]. Similarly, more marked attentional and approach biases towards cannabis cues emerged in those with more severe cannabis use‐related problems [7]. Moreover, cannabis craving is a key symptom of CUD [8] and is an important end‐point when testing the efficacy of treatment [9]. However, the image sets used in individual studies are highly variable and are often developed by individual research teams specifically for their own studies without wider use in the scientific community. Furthermore, the reliability and validity of the images used for cannabis cue reactivity paradigms are often not estimated. This hinders the replicability and quality of evidence generated by individual studies, as well as the evidence synthesis through meta‐analyses. Overall, this evidence points to the importance of using robust paradigms to measure cannabis cue reactivity [10]. To this end, the use of standardized cannabis cues and closely matched control cues is essential [10].

There have been multiple efforts to create databases of cannabis images to measure behavioral, physiological and neural responses to cannabis cues. These efforts have led to the creation of invaluable experimental resources, including the Cannabis Cue Stimulus set [11], Cannabis Images from Australia database [12], Alcohol, Tobacco, and Other Drug Public Domain Photo Database (www.jsad.com/photos), and other image databases [4]. However, some methodological limitations persist. First, the matching of the cannabis and control images is often limited. That is, control images are inadequately matched to the cannabis images regarding size, shape, luminosity and complexity, or image data sets sometimes lack control images entirely [12]. This precludes the use of these images in experimental paradigms that require a closely matched control condition. Second, the content of the images is often heterogenous, for example, by including visual information other than cannabis in the image (e.g. warning signs, people, written language), and the quality of some of the images is relatively low. In those cases, we cannot confirm that participants’ responses are exclusively attributable to cannabis cues. Third, the global representativeness of the images as well as of the participants in the studies is limited, with individual studies reporting data from specific countries, including the USA [11] (www.jsad.com/photos), Australia [12] and Iran [4].

Importantly, there are large regional differences in the diversity and mode of consumption of cannabis products, including the co‐use of cannabis and tobacco [13]. For example, while cannabis combustion is globally the most prevalent route of administration, mixing cannabis with tobacco is more common in Europe (e.g. the Netherlands, 87.6%) than in the USA (4.4%) or Australia (51.6%) [14]. Notably, there is limited research investigating the interacting effects between cannabis and tobacco. Preliminary evidence suggests that mixing cannabis with tobacco is associated with a greater severity of CUD [15, 16], and that tobacco use may mask some effects of cannabis on cognition [17, 18]. On a neural level, differences in cue‐induced brain activity between individuals who use cannabis and controls have also been shown to be affected by an individual’s tobacco use [19], highlighting the importance of accounting for tobacco use when examining cannabis use. To the best of our knowledge, studies including a more globally representative sample and a comparison between individuals who mix or do not mix cannabis with tobacco are lacking.

This study aimed to create a novel, open‐access, standardized and cross‐culturally representative database of cannabis use‐related and control images, matched by resolution, luminosity and complexity: the Cannabis Research Image Database (CRESIDA). Subjective ratings of cue‐induced cannabis craving, tobacco craving, valence and arousal were collected from participants from multiple countries—the USA, the Netherlands and Australia—with variable frequency of cannabis use ranging from monthly to daily. Furthermore, we generated heat maps for each image, illustrating which part of each image captured participants’ attention/focus first to inform future research. The open‐access image database enclosed (https://osf.io/dc9nz/) incorporates ratings of cannabis craving, tobacco craving, valence and arousal, along with the characteristics of participants who rated the images (e.g. cannabis use level, cannabis and tobacco co‐use) to equip cannabis scientists with images they can select based on their specific study aims.

The secondary aim was to investigate the association between subjective cannabis cue‐induced craving and the severity of cannabis use, and if this association was moderated by cannabis image type (e.g. depicting cannabis products, paraphernalia and cannabis use actions). Third, as an illustrative example of how specific sample characteristics can influence cue reactivity, and how this data set can be used to examine cue reactivity in specific subgroups, we explored the role of mixing cannabis with tobacco by comparing cannabis and tobacco craving in response to cannabis images in individuals who mixed cannabis with tobacco versus those who did not, and by testing whether cannabis craving differed depending on cannabis and tobacco co‐use.

## METHODS

### Participants

A total of 1488 individuals who used cannabis at least monthly during the past year participated between January 2022 and May 2024 (USA = 1293; Australia = 43; the Netherlands = 152). In the USA, primary recruitment took place through Amazon’s Mechanical Turk (MTurk) platform and snowballing. In all three countries, participants were recruited online via Instagram, Facebook and cannabis‐oriented Reddit groups, with Gumtree used additionally in Australia. In Australia and the Netherlands, the survey was also sent to previous participants who had consented to future contact. In the Netherlands, recruitment was supplemented by flyer distribution in cannabis outlets and around the university campus. Participants were required to be at least 18 years of age and no older than 64 years, confirm that they have used cannabis monthly during the past year, and reside in the USA, the Netherlands or Australia. The procedures were approved by the Australian Catholic University Human Research Ethics Committee (HREC:2021‐4E), and all participants provided online informed consent prior to study participation. Those who participated through MTurk received a monetary compensation ($7 USD for 20–30 min of participants’ time), whereas no compensation was provided to the other participants.

### Images and rating

A total of 93 cannabis images and 93 neutral images were included in the study. Our international research team consisted of researchers from the USA, the Netherlands, Australia and the UK. The included images present a selection of the image sets originally created in the UK (T.P.F., C.H., V.L.) and in the Netherlands (J.C.) for various local and cross‐cultural cue reactivity studies, selecting those cannabis and control image pairs that would reflect the cannabis use contexts of the USA, Australia and the Netherlands. In the UK, cannabis images were created from existing research materials. In the Netherlands, cannabis images were created from materials bought in cannabis outlets. Cannabis images included actions (e.g. grinding, rolling a joint), cannabis products (e.g. flower bud, grinded flower) or paraphernalia (e.g. pipe, bong). Neutral images were carefully matched with the cannabis images on background, complexity, color, brightness and size to create 93 image pairs matched on image complexity, size, color and brightness (e.g. pencil sharpening, paper clips, green leaves).

Upon seeing the image, participants were asked to click on the element in the picture they noticed first, to create descriptive heat maps of attention/focus per image. Then, participants responded to four questions, with the answers reported on a visual analog scale (VAS; scalar score). These questions assessed: (i) cannabis craving (‘Looking at this picture, how much do you want to use cannabis?’, rating from 0, not at all, to 10, extremely); (ii) arousal (‘Looking at this picture, how excited do you feel?’; rating from 0, calm, to 10, excited); (iii) valence (‘Looking at this picture, how pleasant does the picture look to you?’; rating from 0, very unpleasant, to 10, very pleasant); and (iv) tobacco craving (‘Looking at this picture, how much do you want to use tobacco right now?’; rating from 0, not at all, to 10, extremely).

### Questionnaires

#### Demographics

Participants provided information on their age, gender (i.e. man, woman, other), employment and country of residence at the beginning of the survey.

#### Cannabis use characteristics

Participants reported their age at onset of first cannabis use and monthly cannabis use, the total years they used cannabis at least monthly, and the number of days per week they used cannabis in a typical week. Furthermore, they indicated their preferred method of use, preferred cannabis product, whether they used it for medicinal purposes and whether they mixed their cannabis with tobacco. Finally, the short three‐item version of the Cannabis Use Disorder Identification Test (CUDIT‐SF) was used to assess the severity of cannabis use and related problems [20]. The CUDIT‐SF effectively identifies individuals who are likely to meet the Diagnostic and Statistical Manual of Mental Disorders, Fifth Edition (DSM‐5) criteria for CUD [20].

#### Alcohol and tobacco use

Participants indicated whether they were lifetime and/or current cigarette smokers. The short three‐item version of the Alcohol Use Disorder Identification Test (AUDIT‐C) was used to assess the amount and frequency of alcohol use [21].

### Procedure

Following online consent procedures, participants completed all demographic items and questionnaires before continuing to the image ratings. Then, each participant was randomly assigned one of three image sets (31 pairs, 62 images in total), which were presented in a fixed order. They saw each image, were asked to indicate their first focus point on the image and complete the four VAS scales before moving to the next image. Attention checks were administered via three items, whereby participants were instructed to leave the item blank: (i) ‘To show that you are reading the instructions carefully, please leave this question blank’ with options from ‘never’ to ‘always’; (ii) ‘To show that you are reading the instructions carefully, please leave this question blank’ with three response options (‘yes’, ‘no’ or ‘maybe’); (iii) ‘To show that you are reading the instructions carefully, please leave this question blank’. The total length of time to complete the questionnaires and image ratings was approximately 30 minutes.

### Data analysis

Analyses were not pre‐registered and the results should be considered exploratory.

#### Assessment of data quality

Several checks were conducted to ensure data quality for the 1488 eligible participants who completed the study. First, individuals who failed the first (*n* = 269) or second (*n* = 83) attention check were removed from the data. Second, individuals who reported an earlier age or longer time of (monthly) cannabis use incompatible with their current age (*n* = 246), incompatible answers in ever and current tobacco use (*n* = 57), or age of first use and age of first monthly use (*n* = 43) were removed. Third, those reporting first cannabis use before the age of 10 years were excluded (*n* = 67). Fourth, we reviewed all open answer questions for potential bot responses and excluded those cases (*n* = 35). This resulted in a total sample of 689 (USA = 513, 67% excluded; Australia = 38, 12% excluded; the Netherlands = 138, 9% excluded), divided over the three image batches with almost equal numbers of participants (batch 1, *n* = 223; batch 2, *n* = 229; batch 3, *n* = 237).

#### Image selection and validation

Wilcoxon signed rank tests—as a non‐parametric alternative to the one‐sample *t*‐test—were used to assess whether cannabis images induced above mid‐point cannabis craving, arousal, valence and tobacco craving, whereby mid‐point was a score of 5 on a VAS scale of 0–10. Similarly, we assessed whether neutral images induced below mid‐point cannabis craving, arousal, valence and tobacco craving (with a score of <5 on a scale from 0 to 10). The analysis for tobacco craving was repeated in sub‐groups of individuals who indicated mixing their cannabis with tobacco or not (i.e. simultaneous use). Cannabis images that did not induce above mid‐point cannabis craving (scores of ≤5) and their neutral counterparts were removed from further analysis. Similarly, the neutral images with cannabis craving scores that were above mid‐point (craving scores of >5) were excluded together with their cannabis counterparts. Finally, images for which insufficient data were collected owing to technical failure were removed. Using the quality‐checked, updated image data set, the internal consistency (Cronbach’s alpha, α) of the cannabis craving, arousal, valence and tobacco craving scores was assessed for cannabis and neutral images separately. All the above‐mentioned analyses were performed for each image batch separately and conducted in JASP 0.19.3 [22].

#### Associations between cannabis use characteristics and cannabis cue‐induced craving

Linear mixed models with restricted maximum likelihood estimation and random intercepts to account for repeated measures were used to assess the associations between cannabis use characteristics and cue‐induced cannabis craving scores, and whether these associations were dependent on the type of cannabis image presented. First, we assessed the association between image type (i.e. action, cannabis product, paraphernalia) and cannabis craving. Second, we examined the association between CUDIT‐SF scores and cannabis craving before assessing whether image type moderated this association. Third, we evaluated the association between days per week of cannabis use and cannabis craving before assessing whether image type moderated this association. Fourth, we assessed whether mixing cannabis and tobacco (yes/no), affected cannabis and tobacco craving, before examining whether image type moderated this association. Models were Bonferroni corrected for using three different cannabis use characteristics as predictors of craving (significance threshold: *P* < 0.017, with *P*_Bonf_ indicating original *P*‐values multiplied by three). Sensitivity analyses were conducted to assess whether significant results would hold when adjusting for age and country. All models were estimated using JASP 0.19.3 [22].

## RESULTS

### Sample characteristics

As summarized in Table 1, 55.9% of the 689 participants were female, most of the sample resided in the USA (74.5%) and most participants were employed (10.9% not employed). Participants were between 18 and 64 years old with an average age of 34.39 years (SD = 11.61 years). On average, participants started using cannabis at age 22 years, used cannabis over 3 days a week and had used cannabis for over 11 years, of which almost 11 years were monthly use. Around half of the participants (53.3%) indicated using cannabis for medicinal reasons, and CUDIT‐SF scores were indicative of moderate to severe cannabis use and related problems [20]. Most participants (84.6%) reported lifetime tobacco use, but only 57% consumed tobacco cigarettes. About 60.4% indicated mixing their cannabis with tobacco. AUDIT‐C scores were below the cut‐off for high‐risk alcohol use [21].

**TABLE 1:** Sample characteristics (*n* = 689).

Categorical outcomes	Description	n	Percentage
Gender	Men/Women/Other	385/298/6	43.3/55.9/0.8
Country	USA/the Netherlands/Australia	513/138/38	74.5/20.0/5.5
Employment	Not employed/Full‐time/Part‐time	75/500/114	10.9/72.6/16.5
Cannabis: product type	Strong flower or bud	304	44.1
	Weak flower	157	22.8
	Hash or resin	99	14.4
	Concentrates	53	7.7
	Edibles	69	10.0
	Other	7	1.0
Cannabis: method of use	Joint	247	35.8
	Blunt	51	7.7
	Pipe or other smoking device	176	25.5
	Vaporizer	65	9.4
	Bong or other water‐filtration device	51	7.4
	Dabs	16	2.3
	Hot knife	12	1.7
	Eating it in food	43	6.2
	Drinking it in tea or infusion	25	3.6
	Other	3	0.4
Cannabis: medical use motives	Yes/No	367/322	53.3/46.7
Mix cannabis and tobacco	Yes/No	416/273	60.4/39.6
Lifetime tobacco use	Yes/No	583/106	84.6/15.4
Current tobacco use	Yes/No	393/296	57.0/43.0

Continuous outcomes	Mean (SD)	Median (MAD)	Range
Age, years	34.29(11.61)	32 (7)	18–64
CUDIT‐SF	4.58(3.03)	5 (2)	0–12
Cannabis: days/week	3.54(2.11)	3 (2)	0–7
Cannabis: use years	11.44(9.82)	7 (4)	0–50
Cannabis: years monthly use	10.80(9.62)	7 (4)	0–45
Cannabis: age first use	22.23(7.54)	20 (4)	10–62
AUDIT‐C	4.36(2.47)	5 (2)	0–12

Abbreviations: AUDIT‐C = alcohol use disorder identification test—short form; CUDIT‐SF = cannabis use disorder identification test—short form; MAD = median absolute deviation.

### Images and heat maps

**FIGURE 1:**
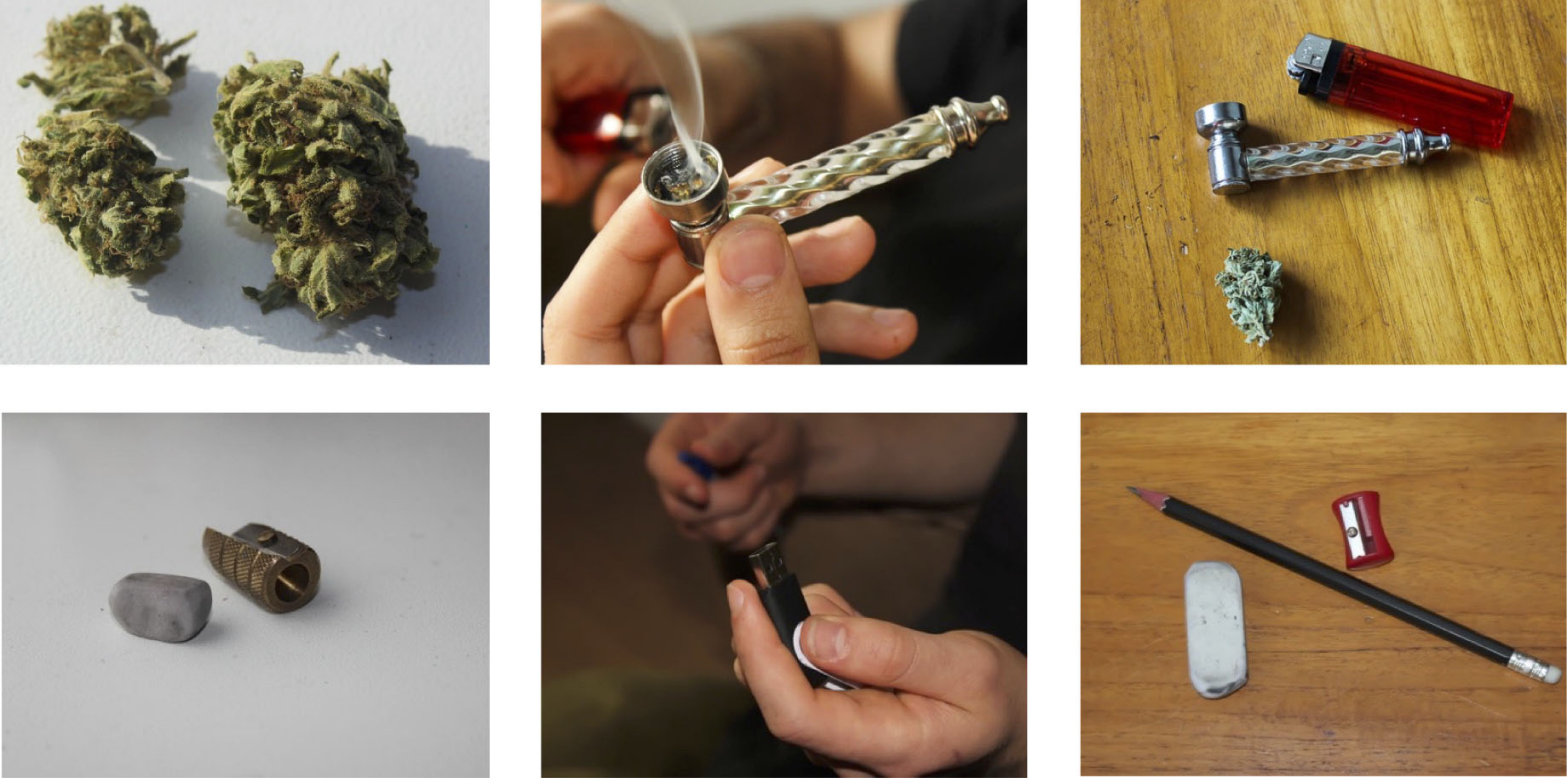
Examples of image types: cannabis product (left), action (middle) and paraphernalia (right).

Figure 1 presents examples of the different image types, including: ‘product’, showing cannabis plant material (left); ‘action’, illustrating the preparation and consumption of cannabis (middle); and ‘paraphernalia’, depicting items commonly used to consume cannabis (right).

**FIGURE 2:**
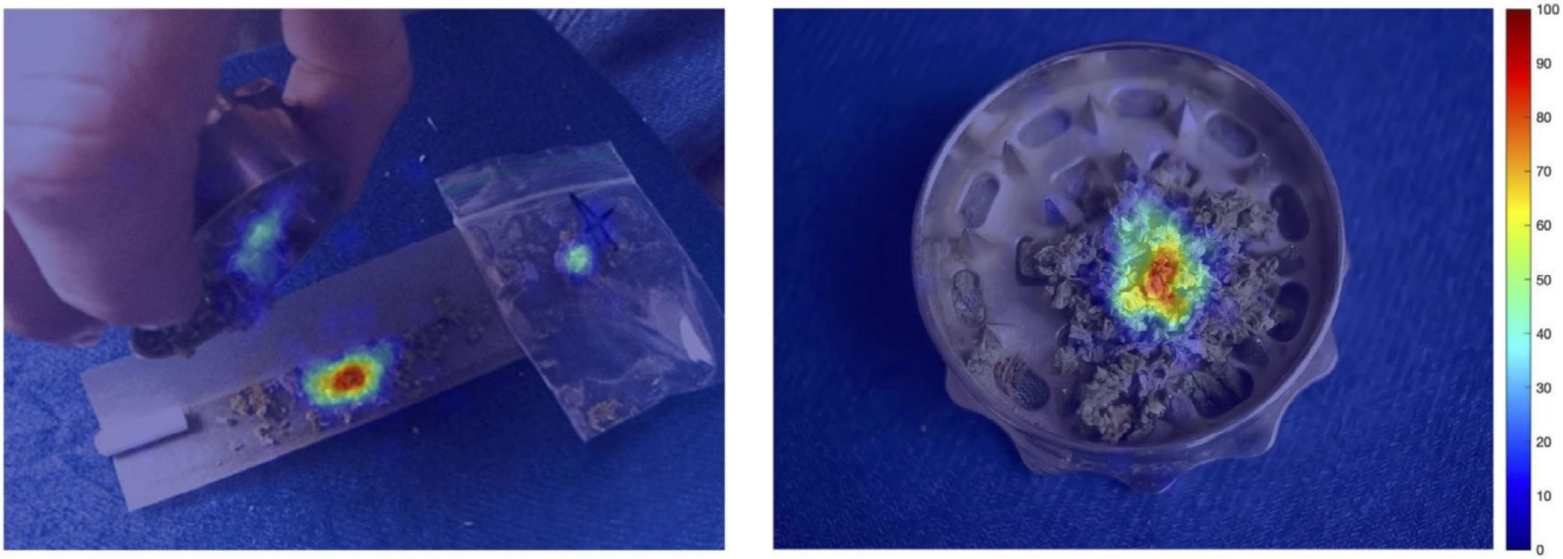
Sample images with heat maps demonstrating a single focal point (right) and multiple focal points (left). The color maps use a range from blue to red, reflecting a low‐to‐high (displayed using a Gaussian function) distribution of focus. The number is normalized by the maximum value of attention/focus, where blue indicates a lower percentage of participants and red indicates a higher percentage of participants.

We generated heat maps for each individual image, demonstrating which part or parts of the image captured participants’ attention/focus (example shown in Figure 2). Color maps use a range from blue to red, reflecting low to high (displayed using a Gaussian function) distribution of focus. The number is normalized by the maximum value of the attention/focus, where blue demonstrates a lower percentage of participants and red indicates a higher percentage of participants. Heat maps for the full data set are provided on the Open Science Framework (OSF) (https://osf.io/dc9nz/). Some images demonstrated single focal points (Figure 2, right), whereas other images attracted multiple focal points (Figure 2, left).

### Image selection and validation

Fifteen image pairs were excluded: 12 cannabis images for failing to show above mid‐point cannabis craving (five cannabis products, seven paraphernalia) and their neutral counterparts; two neutral images for showing above mid‐point cannabis craving and their cannabis counterparts; and one pair owing to technical issues (i.e. item not presented to all participants). This resulted in a final set of 78 image pairs that were used in the following analyses. All images, as well as item‐level summary statistics, are available on the OSF (https://osf.io/dc9nz/).

Looking at the retained image set, the cannabis images—regardless of image type—induced craving and showed above mid‐point (>5) levels of arousal and valence (Table 2). The matched neutral images did not induce craving, showed below mid‐point (<5) levels of arousal and levels at approximately mid‐point (≈5) of valence (Table 2). The cannabis images did not induce tobacco craving in the full sample (Table 3). However, subgroup analyses revealed that cannabis images induced tobacco craving in the group that indicated mixing their cannabis with tobacco (Table 3).

**TABLE 2:** Image ratings of selected image set.

	Cannabis craving		Arousal		Valence	
	M (SD)	Result^a^	M (SD)	Result^a^	M (SD)	Result^a^
**Cannabis images**	6.04(5.25)	>5***	5.71(2.57)	>5***	6.62(1.66)	>5***
Action	6.06(2.51)	>5***	5.73(2.59)	>5***	6.61(1.69)	>5***
Cannabis	6.10(2.60)	>5***	5.71(2.65)	>5***	6.72(1.76)	>5***
Paraphernalia	5.87(2.63)	>5***	5.58(2.67)	>5***	6.50(1.83)	>5***
**Neutral images**	3.45(2.83)	<5***	3.59(2.78)	<5***	4.96(1.99)	ns

Abbreviation: ns = non‐significant.

^a^ One‐sample *t*‐test.

*** *P* < 0.001.

**TABLE 3:** Tobacco craving rating of selected image set in the full sample and subgroups depending on mixing or not mixing cannabis with tobacco.

	Full sample (*n* = 689)		Mix group (*n* = 416)		No‐mix group (*n* = 273)	
	M (SD)	Result^a^	M (SD)	Result^1^	M (SD)	Result^a^
**Cannabis images**	4.19(3.42)	ns	5.66(3.00)	>5***	1.95(2.73)	ns
Action	4.23(3.43)	ns	5.69(3.01)	>5***	1.99(2.76)	ns
Cannabis	4.17(3.46)	ns	5.66(3.03)	>5***	1.90(2.77)	ns
Paraphernalia	4.09(3.34)	ns	5.53(3.06)	>5***	1.90(2.75)	ns
**Neutral images**	3.21(3.05)	<5***	4.39(2.93)	<5***	1.41(2.23)	<5***

Abbreviation: ns = non‐significant.

^a^ One‐sample *t*‐test.

*** *P* < 0.001.

As shown in Table 4, the internal consistency for all image sets—cannabis and neutral images split for each subsection of data—was very high for cannabis craving (lowest α = 0.989), arousal (α = 0.987), valence (α = 0.965) and tobacco craving (α = 0.992).

**TABLE 4:** Internal consistency estimated per section for cannabis and neutral images.

	Section 1				Section 2				Section 3			
	Cannabis		Neutral		Cannabis		Neutral		Cannabis		Neutral	
	α	95% CI	α	95% CI	α	95% CI	α	95% CI	α	95% CI	α	95% CI
Cannabis craving	0.990	0.987–0.993	0.993	0.991–0.994	0.990	0.987–0.993	0.990	0.988–0.993	0.989	0.986–0.992	0.990	0.988–0.992
Arousal	0.988	0.985–0.991	0.991	0.989–0.993	0.988	0.984–0.991	0.987	0.984–0.990	0.987	0.984–0.990	0.989	0.987–0.992
Valence	0.976	0.968–0.983	0.980	0.975–0.984	0.968	0.960–0.976	0.965	0.957–0.973	0.972	0.965–0.979	0.975	0.969–0.980
Tobacco craving	0.995	0.994–0.996	0.995	0.993–0.996	0.995	0.994–0.996	0.992	0.990–0.994	0.995	0.994–0.997	0.992	0.991–0.994

Abbreviation: α = Cronbach’s alpha.

### Associations between cannabis use characteristics and cannabis cue‐induced craving

Image types did significantly affect cannabis craving scores for cannabis images (Table 5, model 1). Bonferroni‐corrected *post hoc* tests using estimated marginal means (EMMs) revealed that this effect was driven by paraphernalia images; the cannabis craving for action images (EMM = 6.062) did not differ from that for cannabis products (EMM = 6.101; *z* = −1.685, *P* = 0.276). However, the cannabis craving for paraphernalia images (EMM = 5.869) was lower than that for images of actions (*z* = 7.128, *P* < 0.001) and for cannabis products (*z* = 7.701, *P* < 0.001).

**TABLE 5:** Associations between cannabis use characteristics and cannabis cue‐induced craving.

Model		Fixed effects estimates				
Image type and craving		B	SE (B)	t	P	P_Bonf_
1	Intercept	6.011	0.096	62.581	<0.001	<0.001
	Image type: action—paraphernalia	0.051	0.013	3.823	<0.001	**<0.001**
	Image type: cannabis—paraphernalia	0.090	0.015	5.846	<0.001	**<0.001**

CUDIT‐SF and craving		B	SE (B)	*t*	*P*	*P*_*Bonf*_
2	Intercept	4.811	0.165	29.201	<0.001	<0.001
	CUDIT‐SF	0.268	0.030	8.935	<0.001	**<0.001**

CUDIT‐SF, image type and craving		B	SE (B)	*t*	*P*	*P*_*Bonf*_
3	Intercept	4.784	0.165	28.987	<0.001	<0.001
	CUDIT‐SF	0.268	0.030	8.915	<0.001	**<0.001**
	Image type: action—paraphernalia	0.049	0.024	2.011	0.044	1.00
	Image type: cannabis—paraphernalia	0.091	0.028	3.248	0.001	**0.003**
	CUDIT‐SF * image type: action—paraphernalia	<0.001	0.004	0.111	0.911	1.00
	CUDIT‐SF * image type: cannabis—paraphernalia	– <0.001	0.005	0.028	0.977	1.00

Day/week and craving		B	SE (B)	*t*	*P*	*P*_*Bonf*_
4	Intercept	5.465	0.186	29.451	<0.001	<0.001
	Days/week	0.162	0.045	3.598	<0.001	**<0.001**

Day/week, image type and craving		B	SE (B)	*t*	*P*	*P*_*Bonf*_
5	Intercept	5.434	0.186	29.137	<0.001	<0.001
	Days/week	0.163	0.045	3.612	<0.001	**<0.001**
	Image type: action—paraphernalia	0.078	0.026	2.994	0.003	0.009
	Image type: cannabis—paraphernalia	0.035	0.030	1.149	0.251	0.753
	Days/week * image type: action—paraphernalia	−0.008	0.006	1.182	0.237	0.711
	Days/week * image type: cannabis—paraphernalia	0.016	0.007	2.144	0.032	0.096

Mixing cannabis with tobacco and craving		B	SE (B)	*t*	*P*	*P*_*Bonf*_
6	Intercept	5.944	0.097	61.563	<0.001	<0.001
	Can/tob mix: no/yes	−0.457	0.097	4.732	<0.001	**<0.001**

Mixing cannabis with tobacco, image type and craving		B	SE (B)	*t*	*P*	*P*_*Bonf*_
7	Intercept	5.915	0.097	61.168	<0.001	<0.001
	Can/tob mix: no/yes	−0.459	0.097	4.744	<0.001	**<0.001**
	Image type: action—paraphernalia	0.050	0.014	3.595	<0.001	**<0.001**
	Image type: cannabis—paraphernalia	0.102	0.016	6.464	<0.001	**<0.001**
	Can/tob mix: no/yes * image type: action—paraphernalia	−0.013	0.014	0.909	0.363	1.00
	Can/tob mix: no/yes * image type: cannabis—paraphernalia	0.055	0.016	3.505	<0.001	**0.001**

*Note*: CUDIT‐SF = cannabis use disorder identification test—short form; can/tob mix = mixing of cannabis with tobacco; Bonf = Bonferroni correction, multiplying *P*‐values by three for comparison with α = 0.05. Significant effects are bolded (except intercept).

Higher CUDIT‐SF scores were associated with higher cannabis craving (Table 5, model 2), but no interaction between CUDIT‐SF and image type was observed (Table 5, model 3). Similarly, the number of days of cannabis use per week was significantly and positively associated with cannabis craving (Table 5, model 4), although no interaction with image type was observed (Table 5, model 5).

**FIGURE 3:**
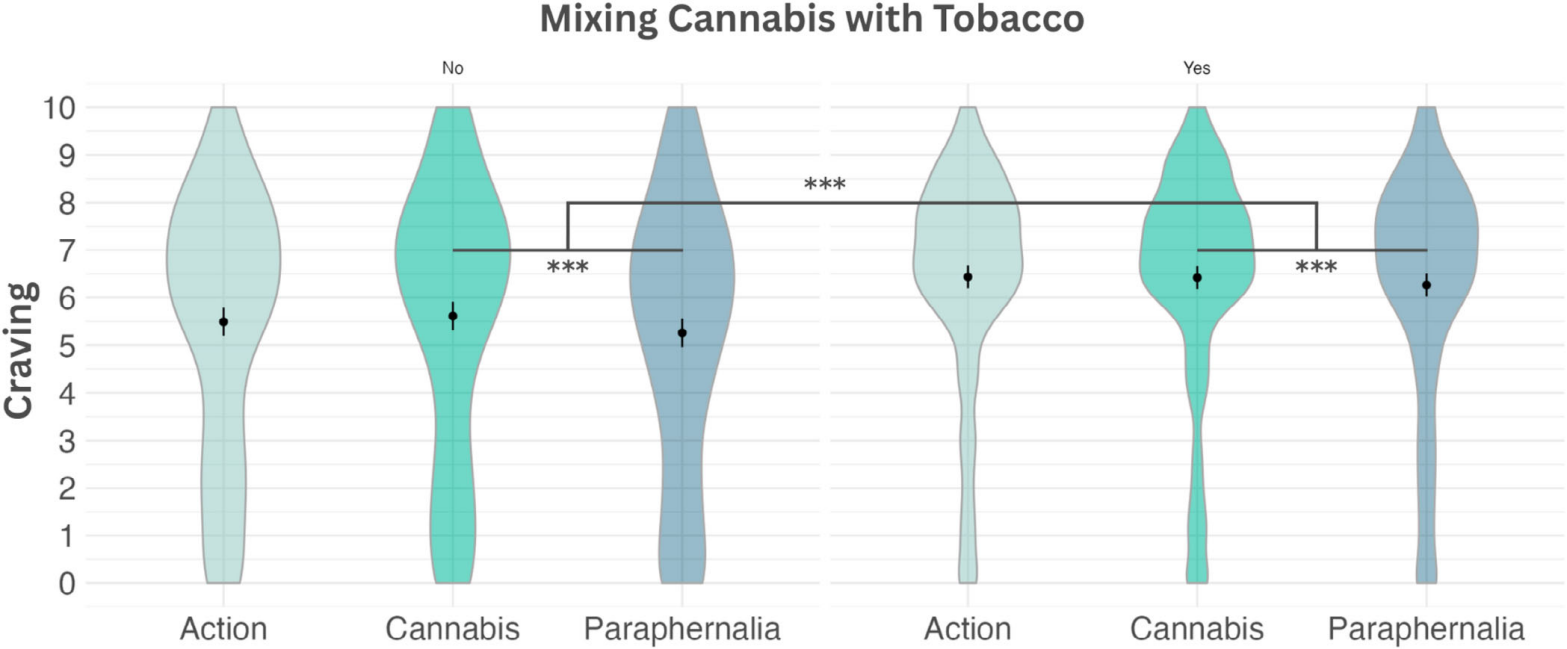
Violin plot visualizing craving per image type for those who mix their cannabis with tobacco (‘Yes’) and those who do not (‘No’). Lines indicate the significant interaction between image type and the mixing of cannabis with tobacco or not. Means are represented with a bar indicating the 95% confidence interval. ***P < 0.001.

Participants who mixed their cannabis with tobacco showed higher cannabis craving (EMM = 6.401) than those who did not (EMM = 5.486; Table 5, model 6). A small but significant interaction between image type and cannabis–tobacco mixing was observed (Table 5, model 7). Bonferroni‐corrected *post hoc* tests using EMMs revealed that in both groups, images of cannabis products induced higher cannabis cravings than images of paraphernalia (mix group, *z* = 4.048, *P* < 0.001; non‐mix group, *z* = 7.265, *P* < 0.001; Figure 3). However, the extent of greater cannabis craving for images of products compared with images of paraphernalia was more marked in participants who consume cannabis alone (paraphernalia, EMM = 5.262; cannabis product, EMM = 5.614; difference, EMM = 0.352; *z* = 3.178, *P* = 0.001) than in those who mixed cannabis with tobacco (paraphernalia, EMM = 6.265; cannabis product, EMM = 6.421; difference, EMM = 0.156). Sensitivity analyses revealed that all linear mixed model results remained similar after adjusting for age and country of residence.

## DISCUSSION

This study aimed to develop and validate the CRESIDA open‐access database of cannabis and closely matched control images. Our validation efforts—including recruiting participants from the USA, the Netherlands and Australia—resulted in a database of 78 cannabis and neutral image pairs, with excellent reliability (α = 0.965–0.995). A greater number of cannabis‐use days per week as well as cannabis‐related problems (CUDIT‐SF) were related to higher cannabis craving in response to cannabis images, regardless of image type (i.e. action, product, paraphernalia). Importantly, individuals who mixed cannabis with tobacco, compared with those who did not, showed the presence of tobacco craving and greater cannabis craving in response to cannabis images, highlighting the importance of considering tobacco–cannabis mixing when studying cannabis craving. All images and the accompanying data are available on the OSF (https://osf.io/dc9nz/). This enables researchers to select validated images that elicit cannabis craving, tailored to their population of interest (e.g. individuals with cannabis and tobacco co‐use, high cannabis use‐related problems, youth, medicinal cannabis users), specific regions or both. The database can also be expanded by adding ratings from additional samples and the integration of new image data sets. For ease of use, the picture database is accompanied by the craving, arousal and valence data aggregated at the image level for the full sample. Furthermore, craving ratings were aggregated for participants who do or do not mix cannabis with tobacco. Moreover, for each individual participant, we provide their subjective ratings (i.e. cannabis craving, tobacco craving, arousal and valence), their socio‐demographic characteristics and their questionnaire total scores.

Out of the original 93 image pairs (i.e. 186 total images), 78 pairs (i.e. 156 total images) were selected based on the craving‐inducing properties of the cannabis images, ensuring their matched neutral counterpart did not induce cannabis craving. The final image database included 21 images of cannabis products (e.g. flower bud, ground flower), 14 images of paraphernalia (e.g. grinder, bong) and 43 images demonstrating cannabis consumption actions (e.g. smoking, rolling a joint). For all cannabis images, self‐reported cannabis craving was high (i.e. >5 out of 10), valence ratings were mildly pleasant (i.e. approximately 5) and arousal was relatively low (i.e. up to 5, out of 10). Craving ratings were slightly higher for images of cannabis products and actions, compared with images of paraphernalia.

In contrast to our findings, earlier work in individuals who use opioids showed higher craving in response to images of opioid paraphernalia relative to those of opioids themselves [23]. This may suggest that cannabis products are more salient cues than paraphernalia for individuals who use cannabis, whereas the opposite pattern may occur among individuals who use opioids, pointing to substance‐specific cue reactivity processes. Greater craving in response to cannabis products versus paraphernalia emerged in both consumers who mix and do not mix their cannabis with tobacco, but this difference was more marked in those who consumed cannabis without tobacco; however, the small effect size limits our ability to draw firm conclusions about whether cannabis–tobacco co‐use differentially influences reactivity to various cannabis image types. Importantly, all subsets of image types showed excellent internal consistency, indicating high reliability of the combined craving estimates. Further adding to the validity of the current image database in inducing cannabis craving, where a higher frequency of cannabis use and a higher level of cannabis use‐related problems were positively associated with craving, regardless of image type.

The consumption of tobacco, both independently from cannabis (e.g. smoking) and simultaneously with cannabis may play a key role in cannabis cue reactivity. Among individuals who smoke cigarettes, those who also vape cannabis showed greater vaping cue‐induced craving than those who do not [24]. Concurrent use is also associated with higher symptoms of CUD [15], potentially also affecting the impact of cannabis on cognition [17], and the underlying neural responses [19]. We extend upon this by showing that cannabis craving was higher in those who mixed cannabis with tobacco compared with those who did not. Furthermore, while in our full sample, none of the images induced tobacco craving; *post hoc* analyses revealed that only the co‐use subsample experienced tobacco cravings in relation to cannabis images. Our findings underscore the challenge of disentangling cannabis‐specific from tobacco‐specific effects and highlight the importance of considering how different patterns of tobacco and nicotine use—whether co‐administered with cannabis or used independently via various methods—may influence cannabis cue reactivity. To this end, accurate and detailed measurement of simultaneous and non‐simultaneous cannabis and tobacco co‐use is critical to understand the mechanisms underlying cannabis and tobacco craving in individuals who use cannabis.

Overall, this study offers several notable strengths, including the creation of an open‐access image database with excellent reliability and validity—including heat maps for each image demonstrating participants’ focal points/attention—and a relatively large sample size. In addition, it features a diverse participant pool: data were collected across three countries, the proportion of medicinal and recreational cannabis users is well balanced, a broad range of ages and consumption levels were included and women were well represented compared with most studies, and were even slightly over‐represented when looking at the global gender differences in use frequency [25]. We provided separate data for individuals who mix or do not mix their cannabis with tobacco. Users of our database can further select their own subsets of participants’ data based on various use and user characteristics or add their own data to our database. However, several limitations should be noted. Online recruitment and data collection have inherent limitations regarding potential fraudulent responses [26], the incorrect reporting of cannabis‐use characteristics and a lack of control over the context in which the study is completed (e.g. concurrent intoxication). Although rigorous attention checks, open text boxes and cross‐checks of cannabis‐related and age‐related questions improved the data quality, we could not verify the accuracy of self‐reported cannabis‐use characteristics and a small number of undetectable ‘imposter’ participants cannot be ruled out. The USA MTurk data had the poorest quality (67% excluded). Moreover, while site differences in cannabis‐use descriptives (Table S1) follow common cross‐cultural trends (e.g. more tobacco use in the Netherlands, more bong and edible use in the USA, later onset age in the USA, and more men than women in the USA and Australia), there are limitations in the regional representativeness of the sample that likely reflect differences in recruitment strategies between sites: for example, the over‐representation of participants from the USA, the under‐representation of Australian participants, and the over‐representation of women and younger participants in the Dutch sample. Nevertheless, to our knowledge, this is the most internationally diverse validation study to date. Interestingly, while use severity did not differ between sites [CUDIT‐SF: *F*(2686) = 0.099, *P* = 0.906], *post hoc* analyses revealed higher cannabis craving scores in the USA sample (M = 6.54) relative to the Australian (M = 5.44, *t* = 2.77, *P*_*Bonf*_ = 0.017) and Dutch (M = 4.36, *t* = 9.59, *P*_*Bonf*_ < 0.001) samples. See Table S2 and Figures S1–S5 for the image ratings per site. Speculatively, these differences may reflect the higher age and longer duration of cannabis use in the USA sample, though future research is needed to verify this. Importantly, regardless of sample imbalances, a clear difference in craving was observed between all cannabis and control images, supporting the validity of the images in cue reactivity research. Moreover, the open‐access database is designed as a living resource to enable researchers to select images suited to specific populations. When using the CRESIDA database, we strongly encourage researchers to contribute their data for others to use. While cannabis flower remains the most used cannabis product in legal and illegal cannabis markets [27], contributing data will enable the database to adapt to changes in cannabis markets internationally (e.g. edibles, vapes, oils). Finally, while craving in response to the cannabis images was related to the frequency of cannabis use and to the severity of cannabis‐related problems, it remains important to validate the current image set and potentially differential reactivity to the different image types in individuals with CUD, confirmed by relevant diagnostic systems, within and outside a treatment context, including youth. Such a study should also randomize the image order to rule out any ordering effects on the craving ratings.

In conclusion, we validated a large, open‐access database of cannabis images and closely matched control images in a multi‐country sample, which can be used and updated by cannabis researchers and clinicians. Cue‐induced ratings of cannabis craving, arousal and valence exhibited excellent reliability, effectively distinguished cannabis images from control images, and were significantly associated with an increased number of cannabis‐use days and cannabis‐related problems. In participants who mixed cannabis and tobacco, compared with those who consumed cannabis alone, the cannabis images elicited tobacco cravings and greater cannabis craving, underscoring the need for future research on cannabis and tobacco co‐use in the study of craving.

## AUTHOR CONTRIBUTIONS

**Janna Cousijn:** Conceptualization; methodology; investigation; data curation; supervision; writing—original draft; writing—review and editing; funding acquisition; project administration. **Emese Kroon:** Methodology; data curation; formal analysis; writing—original draft; writing—review and editing; project administration; validation. **Chao Suo:** Writing—review and editing; visualization. **Tom Freeman P:** Conceptualization; writing—review and editing. **Chandni Hindocha:** Conceptualization; writing—review and editing. **Marianna Quinones‐Valera:** Writing—review and editing; data curation. **Valentina Lorenzetti:** Conceptualization; methodology; investigation; data curation; writing—original draft; writing—review and editing; supervision; funding acquisition; project administration.

## DECLARATION OF INTERESTS

The authors have no conflicts of interest to declare.

## Supporting information


**Figure S1.** Overview of average craving, arousal and valence for neutral images presented per country.
**Figure S2.** Overview of average craving, arousal and valence for cannabis images presented per country.
**Figure S3.** Overview of average craving, arousal and valence for cannabis images—action image subset—presented per country.
**Figure S4.** Overview of average craving, arousal and valence for cannabis images—cannabis image subset—presented per country.
**Figure S5.** Overview of average craving, arousal and valence for cannabis images—paraphernalia image subset—presented per country.
**Table S1.** Sample characteristics per country.
**Table S2.** Overview of image ratings per country for each image type.

## Data Availability

All images and data are available from https://osf.io/dc9nz/ (project DOI: https://doi.org/10.17605/OSF.IO/DC9NZ). Please contact the corresponding authors for further questions regarding data access.
