# Supplementary material for: The Cannabis Research Image Database (CRESIDA): A standardized and validated image set for studying cannabis cue reactivity
Source: Addiction. 2026 Jun 28;121(10):2735–46. doi: 10.1111/add.70516 (PMC13578853; doi:10.1111/add.70516)
Supplement: Supplementary file 1 [file ADD-121-2735-s001.docx]

**Supplementary Materials**

The Cannabis Research Image Database (CRESIDA): an internationally standardized and validated image set for studying cannabis cue-reactivity

Janna Cousijn^1*^, Emese Kroon^1*^, Chao Suo^2,3,4^ Tom P Freeman^5^, Chandni Hindocha^6^, Marianna Quinones-Valera^3^, Valentina Lorenzetti^3^

^1^Neuroscience of Addiction (NofA) lab, Center for Substance Use and Addiction Research (CESAR), Department of Psychology, Education & Child Studies, Erasmus University Rotterdam, The Netherlands

^2^QIMR Berghofer, Queensland, Australia

^3^Neuroscience of Addiction and Mental Health Programe, Healthy Brain and Mind Research Centre, School of Behavioural and Health Sciences, Faculty of Health Sciences, Australian Catholic University, Australia

^4^Turner Institute for Brain and Mental Health, School of Psychological Sciences & Monash

Biomedical Imaging Facility, Monash University, Victoria, Australia

^5^Addiction and Mental Health Group, Department of Psychology, University of Bath, UK

^6^Clinical Psychopharmacology Unit, Research Department of Clinical, Educational & Health Psychology, University College London

**Correspondence:** Janna Cousijn; [cousijn@essb.eur.nl](about:blank); Erasmus University Rotterdam, Department of Psychology, Education & Child Studies, Burgemeester Oudlaan 50, Rotterdam Zuid-Holland, Netherlands; Prof. Valentina Lorenzetti, PhD; [valentina.lorenzetti@acu.edu.au](mailto:valentina.lorenzetti@acu.edu.au); Neuroscience of Addiction and Mental Health Program, Healthy Brain and Mind Research Centre, School of Behavioural and Health Sciences, Faculty of Health Sciences, Australian Catholic University, Level 5 Daniel Mannix Building, 115 Victoria Parade, Fitzroy VIC 3065, Australia

* Authors contributed equally to this work.

| Table S1. Sample characteristics per country | | | | | | | | | |
| --- | --- | --- | --- | --- | --- | --- | --- | --- | --- |
| Categorical Outcomes | **Description** | **Total** | | **USA** | | **NL** | | **Australia** | |
|  |  | **N(%)** | | **N(%)** | | **N(%)** | | **N(%)** | |
| Gender | Men | 385(43.3) | | 310(60.4) | | 51(37.0) | | 24(63.2) | |
|  | Women | 298(55.9) | | 200(39.0) | | 86(62.3) | | 12(31.6) | |
|  | Other | 6(0.8) | | 3(0.6) | | 1(0.7) | | 2(5.3) | |
| Employment | Not employed | 75(10.9) | | 12(2.3) | | 58(42.0) | | 5(13.2) | |
|  | Full-time | 500(72.6) | | 474(92.4) | | 8(5.8) | | 18(47.4) | |
|  | Part-time | 114(16.5) | | 27(5.3) | | 72(52.2) | | 15(39.5) | |
| Cannabis: product type | Strong flower or bud | 304(44.1) | | 196(38.2) | | 80(58.0) | | 28(73.7) | |
|  | Weak flower | 157(22.8) | | 136(26.5) | | 17(12.3) | | 4(10.5) | |
|  | Hash or resin/ | 99(14.4) | | 62(12.1) | | 37(26.8) | | 0(0) | |
|  | Concentrates | 53(7.7) | | 53(10.3) | | 0(0) | | 0(0) | |
|  | Edibles | 69(10.0) | | 63(12.3) | | 3(2.2) | | 3(7.9) | |
|  | Other | 7(1.0) | | 3(0.6) | | 1(0.7) | | 3(7.9) | |
| Cannabis: method of use | Joint | 247(35.8) | | 112(21.8) | | 124(89.9) | | 11(28.9) | |
|  | Blunt | 51(7.7) | | 44(8.6) | | 3(2.2) | | 4(10.5) | |
|  | Pipe or other smoking device | 176(25.5) | | 172(33.5) | | 2(1.4) | | 2(5.3) | |
|  | Vaporizer | 65(9.4) | | 54(10.5) | | 2(1.4) | | 9(23.7) | |
|  | Bong or other water filtration device | 51(7.4) | | 37(7.2) | | 4(2.9) | | 10(26.3) | |
|  | Dabs | 16(2.3) | | 16(3.1) | | 0(0) | | 0(0) | |
|  | Hot knife | 12(1.7) | | 12(2.3) | | 0(0) | | 0(0) | |
|  | Eating it in food | 43(6.2) | | 39(7.6) | | 3(2.2) | | 1(2.6) | |
|  | Drinking it in tea or infusion | 25(3.6) | | 25(4.9) | | 0(0) | | 0(0) | |
|  | Other | 3(0.4) | | 2(0.4) | | 0(0) | | 1(2.6) | |
| Cannabis: medical use motives | Yes | 367(53.3) | | 326(63.5) | | 17(12.3) | | 24(63.2)) | |
|  | No | 322(46.7) | | 187(36.5) | | 121(87.7) | | 14(36.8) | |
| Mix cannabis and tobacco | Yes | 416(60.4) | | 283(55.2) | | 117(84.8) | | 16(42.1) | |
|  | No | 273(39.6) | | 230(44.8) | | 21(15.2) | | 22(57.9) | |
| Lifetime tobacco use | Yes | 583(84.6) | | 423(82.5) | | 127(92.0) | | 33(86.8) | |
|  | No | 106(15.4) | | 90(17.5) | | 11(8.0) | | 5(13.2) | |
| Current tobacco use | Yes | 393(57.0) | | 318(62.0) | | 66(47.8) | | 9(23.7) | |
|  | No | 296(43.0) | | 195(38.0) | | 72(52.2) | | 29(76.3) | |
| Continuous Outcomes | | **M(SD)** | **Med(MAD)** | **M(SD)** | **Med(MAD)** | **M(SD)** | **Med(MAD)** | **M(SD)** | **Med(MAD)** |
| Age | | 34.29(11.61) | 32(7) | 37.73(10.80) | 34(6) | 21.96(3.26) | 21(2) | 32.50(10.84) | 29(7) |
| CUDIT-R short | | 4.58(3.03) | 5(2) | 4.61(2.92) | 5(2) | 4.49(3.40) | 4(3) | 4.5(3.19) | 4(2) |
| Cannabis: days/week | | 3.54(2.11) | 3(2) | 3.50(1.96) | 3(1) | 3.35(2.74) | 2(1) | 4.49(2.40) | 5.5(1.5) |
| Cannabis: use years | | 11.44(9.82) | 7(4) | 13.17(10.19) | 10(5) | 4.70(3.60) | 4(1) | 12.61(9.79) | 9(6) |
| Cannabis: years monthly use | | 10.80(9.62) | 7(4) | 12.77(9.86) | 10(6) | 3.83(3.55) | 3(1) | 9.50(9.51) | 5.5(3.5) |
| Cannabis: age first use | | 22.23(7.54) | 20(4) | 22.80(7.99) | 21(4) | 16.35(2.21) | 16(1) | 17.57(4.46) | 16(1) |
| AUDIT short | | 4.36(2.47) | 5(2) | 4.20(2.51) | 5(2) | 5.04(2.68) | 5(2) | 4.08(2.71) | 4(2) |
| Note. AUDIT: alcohol use disorder identification test; CUDIT-R: cannabis use disorder identification test-revised; Med: Median; MAD: median absolute deviation; M: mean; SD: standard deviation. | | | | | | | | | |

| **Table S2. Overview of image ratings per country for each image type** | | | | | | | |
| --- | --- | --- | --- | --- | --- | --- | --- |
| **Image Type** | **Outcome** | **Country** | **N** | **Median** | **IQR** | **Mean** | **SD** |
| Neutral | Craving | Australia | 38 | 1.101 | 3.037 | 2.112 | 2.712 |
|  |  | Netherlands | 138 | 1.095 | 2.964 | 1.701 | 1.699 |
|  |  | United States | 513 | 3.964 | 5.250 | 4.024 | 2.861 |
|  | Arousal | Australia | 38 | 1.339 | 2.822 | 2.121 | 2.496 |
|  |  | Netherlands | 138 | 1.712 | 2.768 | 1.925 | 1.654 |
|  |  | United States | 513 | 4.071 | 5.109 | 4.145 | 2.820 |
|  | Valence | Australia | 38 | 4.542 | 1.804 | 4.452 | 1.897 |
|  |  | Netherlands | 138 | 3.853 | 2.204 | 3.562 | 1.453 |
|  |  | United States | 513 | 5.321 | 2.602 | 5.371 | 1.948 |
| Cannabis –  All Type | Craving | Australia | 38 | 5.797 | 3.512 | 5.436 | 2.916 |
|  |  | Netherlands | 138 | 4.875 | 4.940 | 4.363 | 2.954 |
|  |  | United States | 513 | 6.857 | 1.968 | 6.536 | 2.129 |
|  | Arousal | Australia | 38 | 5.404 | 5.243 | 4.969 | 3.070 |
|  |  | Netherlands | 138 | 4.619 | 4.732 | 4.237 | 2.812 |
|  |  | United States | 513 | 6.625 | 2.304 | 6.154 | 2.298 |
|  | Valence | Australia | 38 | 6.572 | 2.092 | 6.592 | 1.681 |
|  |  | Netherlands | 138 | 5.963 | 2.145 | 5.745 | 1.957 |
|  |  | United States | 513 | 6.833 | 1.791 | 6.856 | 1.492 |
| **Cannabis Image Type** | **Outcome** | **Country** | **N** | **Median** | **IQR** | **Mean** | **SD** |
| Action | Craving | Australia | 38 | 5.850 | 3.507 | 5.422 | 2.901 |
|  |  | Netherlands | 138 | 4.841 | 5.074 | 4.492 | 2.967 |
|  |  | United States | 513 | 6.941 | 2.085 | 6.533 | 2.143 |
|  | Arousal | Australia | 38 | 5.824 | 5.037 | 4.915 | 3.092 |
|  |  | Netherlands | 138 | 4.724 | 4.625 | 4.354 | 2.827 |
|  |  | United States | 513 | 6.636 | 2.433 | 6.165 | 2.326 |
|  | Valence | Australia | 38 | 6.238 | 2.136 | 6.476 | 1.772 |
|  |  | Netherlands | 138 | 5.954 | 2.367 | 5.852 | 2.004 |
|  |  | United States | 513 | 6.800 | 1.882 | 6.829 | 1.533 |
| Cannabis | Craving | Australia | 38 | 6.366 | 4.135 | 5.549 | 3.021 |
|  |  | Netherlands | 138 | 5.000 | 5.601 | 4.295 | 3.050 |
|  |  | United States | 513 | 7.000 | 2.000 | 6.623 | 2.173 |
|  | Arousal | Australia | 38 | 5.405 | 5.240 | 5.172 | 3.147 |
|  |  | Netherlands | 138 | 4.167 | 5.036 | 4.108 | 2.919 |
|  |  | United States | 513 | 6.667 | 2.339 | 6.184 | 2.337 |
|  | Valence | Australia | 38 | 7.358 | 2.541 | 7.037 | 1.760 |
|  |  | Netherlands | 138 | 6.000 | 2.778 | 5.734 | 2.113 |
|  |  | United States | 513 | 7.000 | 1.958 | 6.964 | 1.558 |
| Paraphernalia | Craving | Australia | 38 | 5.700 | 4.375 | 5.219 | 2.932 |
|  |  | Netherlands | 138 | 4.286 | 5.350 | 4.019 | 2.917 |
|  |  | United States | 513 | 6.857 | 2.357 | 6.415 | 2.268 |
|  | Arousal | Australia | 38 | 5.357 | 5.050 | 4.729 | 3.014 |
|  |  | Netherlands | 138 | 4.343 | 4.900 | 3.970 | 2.806 |
|  |  | United States | 513 | 6.571 | 2.714 | 6.073 | 2.413 |
|  | Valence | Australia | 38 | 6.143 | 2.503 | 6.247 | 1.798 |
|  |  | Netherlands | 138 | 5.500 | 2.447 | 5.439 | 2.140 |
|  |  | United States | 513 | 6.857 | 2.000 | 6.799 | 1.620 |

**Figure S1. Overview of average craving, arousal and valence for neutral images presented per country.** Diamond represents the mean.

**Figure S2. Overview of average craving, arousal and valence for cannabis images presented per country.** Diamond represents the mean.

**Figure S3. Overview of average craving, arousal and valence for cannabis images – action image subset - presented per country.** Diamond represents the mean.

**Figure S4. Overview of average craving, arousal and valence for cannabis images – cannabis image subset - presented per country.** Diamond represents the mean.

**Figure S5. Overview of average craving, arousal and valence for cannabis images – paraphernalia image subset - presented per country.** Diamond represents the mean.
